# Grindability of carbon fiber reinforced polymer using CNT biological lubricant

**DOI:** 10.1038/s41598-021-02071-y

**Published:** 2021-11-18

**Authors:** Teng Gao, Yanbin Zhang, Changhe Li, Yiqi Wang, Qinglong An, Bo Liu, Zafar Said, Shubham Sharma

**Affiliations:** 1 Qingdao University of Technology, Qingdao, 266520 China; 2 Dalian University of Technology, Dalian, 116024 China; 3 Shanghai Jiao Tong University, Shanghai, 200240 China; 4 Sichuan Future Aerospace Industry LLC., Shifang, 618400 China; 5 University of Sharjah, 27272 Sharjah, United Arab Emirates; 6 IK Gujral Punjab Technical University, Punjab, 144603 India

**Keywords:** Mechanical engineering, Other nanotechnology, Aerospace engineering

## Abstract

Carbon fiber-reinforced polymer (CFRP) easily realizes the integrated manufacturing of components with high specific strength and stiffness, and it has become the preferred material in the aerospace field. Grinding is the key approach to realize precision parts and matching the positioning surface for assembly and precision. Hygroscopicity limits the application of flood lubrication in CFRP grinding, and dry grinding leads to large force, surface deterioration, and wheel clogging. To solve the above technical bottleneck, this study explored the grindability and frictional behavior of CNT biological lubricant MQL through grinding experiments and friction-wear tests. Results showed that the CNT biological lubricant reduced the friction coefficient by 53.47% compared with dry condition, showing optimal and durable antifriction characteristics. The new lubrication was beneficial to suppressing the removal of multifiber block debris, tensile fracture, and tensile-shear fracture, with the advantages of tribological properties and material removal behavior, the tangential and normal grinding force, and the specific grinding energy were reduced by 40.41%, 31.46%, and 55.78%, respectively, compared with dry grinding. The proposed method reduced surface roughness and obtained the optimal surface morphology by preventing burrs, fiber pull-out, and resin smearing, and wheel clogging was prevented by temperature reduction and lubricating oil film formation. *S*_a_ and *S*_q_ of the CNT biological lubricant were reduced by 8.4% and 7.9%, respectively, compared with dry grinding. This study provides a practical basis for further application of CNT biological lubricant in CFRP grinding.

## Introduction

Carbon fiber-reinforced polymer (CFRP) has become the preferred material for aerospace equipment due to its high specific strength, high specific stiffness, and integrated design and manufacturing of material structure, and good performance^1,2^. Thus, CFRP can easily realize the integrated manufacturing of large and complex components, greatly reduce connections and weight, and improve performance and reliability^3^. At present, near net shape manufacturing of CFRP is mainly achieved by laying and curing^4^. However, to ensure the accuracy of components and assembly requirements, a large amount of secondary processing of edge profiles, function windows, and connecting holes is still needed to assemble composite parts after curing^5,6^. Delamination and other damages in the process of machining and assembly will seriously affect the bearing capacity of components and threaten service safety^7^. Therefore, CFRP must be processed with high quality and low damage^8^. CFRPs are usually large and thick, resulting in a large amount of material removal, which is why cutting process is still the main processing method^9^. Moreover, with the gradual expansion of the application field of CFRPs, the increasingly stringent requirements have posed greater challenges to the applicability of materials^10^. The processing quality requirements are also becoming increasingly strict, thereby resulting in higher processing costs^11^. Generally, in the aerospace field, the machining quality of CFRPs is higher. The surface quality and machining accuracy of the workpiece need to be in a higher processing mode, and grinding and precision grinding processes must meet manufacturing requirements^12–15^. Currently, grinding through diamond abrasives is still the major conventional machining method applied to brittle materials^16,17^. The main structures of CFRP are always subjected to ultimate loads, thus requiring strict tolerances for machining damage^18^. However, the processing properties of CFRP mainly depend on the properties of fiber and matrix and their comprehensive influence on the damage mechanism^19,20^. The machining damage essentially depends on the formation mechanism of the chips in the machining process^21^. At present, basic research of CFRP is still a hot issue in the modern manufacturing industry.

Su et al.^22^ observed fibers with strong constraint fracture at a local contact area, such as at 45°. However, this issue becomes a combination of the bending and the local contact due to the large bending deformation with the decrease in the off-axis modulus along the cutting direction of 90°. Furthermore, the bending-dominant chip formation easily results in severe subsurface damage due to the large bending deformation. Lasri et al.^23^ simulated the chip formation process by using the stiffness degradation concept and appropriate failure criteria, and analyzed the cutting damage from chip initiation to complete chip formation. Debonding of fiber and matrix is the first failure mode in CFRP cutting. It germinates near the cutting edge of the tool and is accompanied by matrix cracking at different stages of chip formation. Fiber fracture is the last failure mode in the process of chip formation. Zenia et al.^24^ developed a finite element model to analyze the chip formation process and subsurface damage. They found that 90° fiber orientation is the critical orientation that generates severe damage. On the basis of the investigation of chip formation, an approach for predicting fracture toughness of the newly machined surface was proposed. Results showed that the proportion of energy spent on tool-chip friction was the greatest, and the proportions of energy spent on creating a new surface decrease as the fiber angle increases^25^. Agarwal et al.^26^ found that the chip formation mechanism in FRP depends on the cutting depth and fiber orientation, and pure epoxy resin shows a significant size effect. Rao et al.^27^ found that the degradation of the matrix adjacent to the fiber occurs first, followed by failure of the fiber at its rear side. The extent of sub-surface damage due to matrix cracking and interfacial debonding was also determined. Existing research shows that the type of the chip formation was highly dependent on the fiber orientation and the rake angle. Fiber orientation (*θ*) affects the chip formation significantly^28^. Fiber fracture above the trimming path dominates chip formation. Chipping occurred mainly through bending-induced fracture (*θ* < 90°) and crushing fracture (*θ* > 90°) of fibers. Compared with a traditional cutting process, elliptical vibration assist cutting can minimize the fiber orientation effect through localized fiber fracture. The failure mode of fiber was similar to that of micro-buckling, and the interface debonding will not extend to the subsurface when *θ* is 90°^29^. With the increase in the ratios of cutting depth to the cutting edge diameter, the failure mode turns into kink band formation. Ohashi et al.^30^ discussed the effects of water-soluble coolant and liquid nitrogen on the grinding characteristics of CFRP compared with dry grinding. Liquid nitrogen has a certain effect on preventing wheel loading and carbon fiber delamination. However, the hardness of CFRP increases due to solidification, and the grinding force provided by liquid nitrogen was greater than that provided by dry grinding. Kodama et al.^31^ found that the temperature in the contact zone of the grinding wheel increased to the thermal decomposition temperature of matrix resin during dry grinding. In wet conditions, the grinding temperature will not rise until the thermal decomposition temperature is reached. The thermal decomposition of the matrix resin leads to the decrease in the surface finish. The micro-damage of carbon fiber occurs during wet grinding.

Minimum quantity lubrication (MQL) between dry and flood lubrication involves mixing high-pressure gas with minute quantities of lubricant to form micro droplets, which are sprayed on the grinding area at a high speed through a nozzle for effective cooling and lubrication^32,33^. The MQL fluid forms a lubricating oil film at the interface between the grinding wheel and workpiece, thus playing a lubricating role^34,35^. High-pressure air mainly plays the roles of cooling and debris removal. To realize cleaner production from the source of machining, vegetable oil with high biodegradability and nontoxicity is generally selected as the base oil of MQL fluid^36,37^, which greatly reduces the harm of cutting fluid to the environment and the human body^38^. The flow rate of MQL is generally 30–100 ml/h, which is only 0.1% of the traditional flood lubrication (60 L/h). However, the ability of MQL to remove heat in the grinding zone mainly relies on high-pressure gas, thus being unable to achieve the expected cooling effect and leading to the deterioration of the surface integrity^39–41^. Nanofluid minimum quantity lubrication (NMQL) is a new cooling and lubrication technique with clean, low consumption and high efficiency^42^. The technical approach is to add one or several kinds of nanoparticles that have different physical and chemical properties to MQL base oil, thus making the nanoparticles disperse and mix to form nanofluids^43,44^. Then, the nanofluids are atomized under high-pressure air flow and then sprayed on the grinding area through a nozzle^45^. NMQL effectively solves the problem of insufficient heat transfer capacity of MQL in the grinding zone and improves the lubrication performance of the interface between the grinding wheel and the workpiece^46,47^. In addition, nanoparticles can improve the lubrication characteristics of grinding wheel/workpiece and grain/chip interfaces due to their excellent tribological properties^48^. Yang et al.^49^ found that both minimum chip thickness and ductile–brittle transition chip thickness decreased with increasing friction coefficient in the grinding of zirconia ceramics. In accordance with the trend of the specific energy of single grain and the unit grinding force, the position where the size effect occurs was determined^50^. The size effect mainly occurred in the plowing area. Gao et al.^51^ proved that compared with dry grinding, NMQL grinding CFRP can obtain lower surface roughness and fractal dimension, and effectively reduce processing damage such as resin coating, multifiber block pulling-out, and pits. Rodriguez et al.^52^ used optimized lubrication (high pressure, low flow, semi-synthetic double oil), MQL, and dry grinding as alternatives to traditional coolant technology. The surface grinding behavior of CFRP was analyzed by using scanning electron microscopy (SEM) images of the workpiece surface. Results show that MQL has the lowest grinding value and specific grinding energy. Xu et al.^53^ established a new simulation tool chip slit with micron geometry to evaluate and quantify the permeability of nanographite nanofluids. In the closed broaching process, nanofluids can penetrate the chip slot and obtain excellent lubrication performance.

The above literature review indicates that the dry grinding CFRP was most commonly used. However, CFRP is sensitive to temperature and easily causes thermal damage, thereby leading to a series of induced processing damage and deterioration of surface quality. Carbon nanotube (CNT) biological lubricant MQL is an efficient new green cooling and lubrication technology. CNTs have high thermal conductivity, which is beneficial to removing heat from the grinding zone. At present, little research has been conducted on CNT biological lubricant-assisted grinding CFRP. In this paper, CNTs were added to palm oil to prepare a biological nano-lubricant for CFRP grinding, and single grain and grinding wheel experiments were conducted. The grindability of the new lubrication method was evaluated from the aspects of friction-wear behavior, chip and groove morphology, grinding force, specific grinding energy, surface roughness, three-dimensional surface topography, and wheel clogging. This work provides theoretical and practical basis for further research and application of CNT nano-lubricant in CFRP grinding.

## Experimental

### Grinding experiments of single grain and grinding wheel

**Figure 1:**
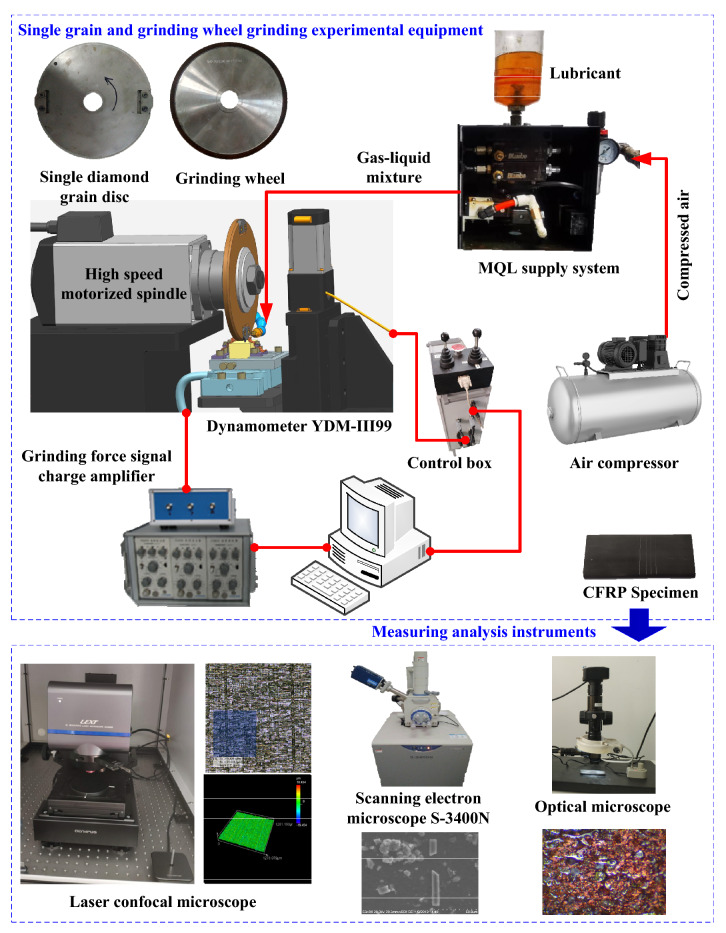
Single grain and grinding wheel grinding experimental equipment and measuring analysis instruments. Figures were created using the Microsoft Visio sofware (version 2010).

Research on the grinding mechanism can start from single grain grinding, integrate the cutting results of single grain in the grinding area effectively, and then explain the physical phenomena that took place during the grinding process. Single grain grinding is a simplified analysis method for complex grinding processes, which are different from the actual grinding process. Single grain grinding has a certain reference value for material removal, chip formation mechanism, and grain wear. The grinding experimental platform is shown in Fig. 1. The diamond grain has a size of about 550 μm and was fixed on the insert of the grinding wheel disc through brazing. The material properties of single grain and grinding wheel are shown in Table 1. A three-phase dynamometer YDM-III99 was clamped on the platform to collect force signals during the grinding process. The grinding force of each group was measured in five groups, and the average value was used. The MQL device in the experiment was the Bluebee MQL system, which uses a pulse generator to adjust the CNT nano-lubricant and compressed gas in the MQL system, thus controlling their flow. The proper gas–liquid ratio can be selected for processing. The grinding experiment parameters of single grain and grinding wheel are shown in Table 2. The compressed gas and CNT nano-lubricant were mixed and atomized, and then they were ejected from the nozzle with the assistance of high-pressure gas into the grinding area. The MQL system includes adjustable flow pumps, compressed air flow control devices, and spray-assisted air atomizing nozzles.

**Table 1:** Material properties of polycrystalline diamond single grain and grinding wheel.

Properties	Value
**Single diamond grain**	
Density (× 10^3^ kg/m^3^)	4.12
Hardness (GPa)	50.00
Young’s modulus (GPa)	776.00
Poisson’s ratio	0.07
Transverse fracture strength (GPa)	1.20
**Grinding wheel**	
Grain materials	Diamond
Binding agent	Resin
Granularity	170 #

**Table 2:** Single grain and grinding wheel grinding experiment parameters.

Grinding experiment parameters	Value
Peripheral speed of grinding wheel *v*_s_ (m/s)	30
Depth of cut *a*_p_ (μm)	20
Feed speed *v*_*w*_ (mm/s)	30
Grinding wheel diameter (mm)	200
Gas pressure (bar)	6.0
Gas–liquid ratio	3.0
MQL flow rate (mL/h)	60
MQL nozzle distance (mm)	12
MQL nozzle angle (°)	15

### Friction-wear test

**Figure 2:**
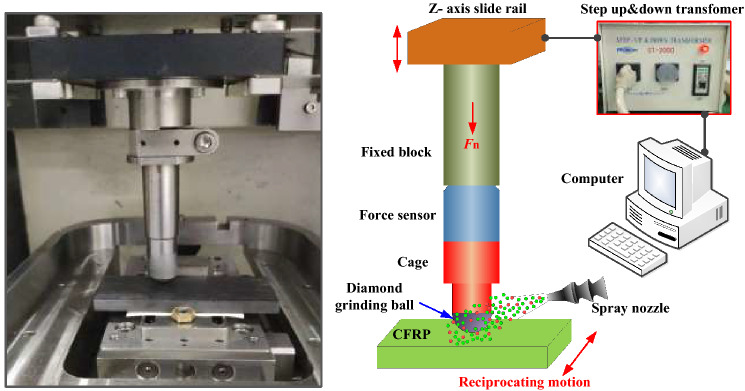
UMT-3 friction and wear tester. Figures were created using the Microsoft Visio sofware (version 2010) and SOLIDWORKS (version 2018).

The friction–wear test needs to be performed under conditions similar to grinding conditions^54^. As shown in Fig. 2, the UMT-3 tester produced by CETR was used to characterize the tribological properties under different lubrication conditions. The system has a variety of modules that can be directly replaced, and it can simulate a variety of experimental conditions of friction pair motion, such as point-to-point contact and point-to-face contact. The system also has a variety of replaceable modules, such as ball on disc, pin on disc, and ring block model, and reciprocating and rotary motion modes, among others. The selection of test parameters is shown in Table 3. The data of vertical loads and horizontal frictions collected by the top two channel force sensors were fed back to the computer and recorded in real time. The ratio of frictions and loads is the friction coefficient, and then the relationship curve of the friction coefficient with time was obtained.

**Table 3:** Friction test parameters.

Test conditions	Value
Contact condition of friction pair	Point-to-face contact
Module type	Pin (diamond)–disk (CFRP) model
Mode of motion	Reciprocating
Test temperature (°C)	22 ± 5
Test duration (s)	1200
Load (N)	10
Frequency (Hz)	50

### Methods and materials

The size of the CFRP workpiece was 70 mm × 30 mm × 6 mm, and the material properties are shown in Table 4. The experiment was performed in three lubri-cooling modes: dry, MQL, and CNT nano-lubricant MQL. The experimental scheme is shown in Table 5. The MQL lubricant was a CNT nano-lubricant with the volume fraction of nanoparticles of 2 vol%, and the base oil was palm oil. The fatty acids of palm oil are mainly palmitic acid (about 30–50%), part of stearic acid is saturated fatty acids, and the number of C atoms is 16. Therefore, a lubricating film with strong adsorption force can be formed to achieve good lubrication performance. This was the reason palm oil was chosen as the base oil of biological-based nano-lubricant. For the same kind of solid materials with the same mass, a small volume and particle size corresponds to a large specific surface area, thus resulting in excellent thermal conductivity. CNTs were chosen as nano-additive because of their excellent tribological properties and thermal conductivity^55^. CNTs’ constraints of winding and conglobations contradict their tribological performance, but they can be solved by adding surfactants^56^. Therefore, the surfactant APE-10 was added to the CNT nano-lubricant.

**Table 4:** Specimen material properties.

**CFRP**	
Density (g/cm^3^)	1.575
Hardness (HRB)	68–72
Transverse elasticity modulus (GPa)	9.65
**Interface**	
Interfacial bonding strength (MPa)	30
Interfacial equivalent modulus *k*_b_ (GPa/m)	115
**Carbon fiber (70%.vol)**	
Density (g/cm^3^)	1.8
Young’s modulus (GPa)	230
Transverse elasticity modulus (GPa)	19.6
Axial tensile strength (GPa)	3.45
Shear strength (GPa)	0.38
Poisson’s ratio	0.3
Fracture toughness (J/m^2^)	2
Elongation at break (%)	1.5
Monofilament diameter (μm)	7.0
**Vinyl resin (30%.vol)**	
Density (g/cm^3^)	1.05
Young’s modulus (GPa)	3.4
Axial tensile strength (MPa)	85
Shear strength GPa)	1.02
Poisson’s ratio	0.4
Fracture toughness (J/m^2^)	500
Elongation at break (%)	5.5
Heat deflection temperature (HDT) (℃)	101

**Table 5:** Experimental scheme.

Lubricating conditions	Lubricant
Dry grinding	n/a
MQL	Pure palm oil
CNT nano-lubricant MQL	Pure palm oil-based CNT (50 nm) nano-lubricant

## Results and discussion

### Frictional behavior

**Figure 3:**
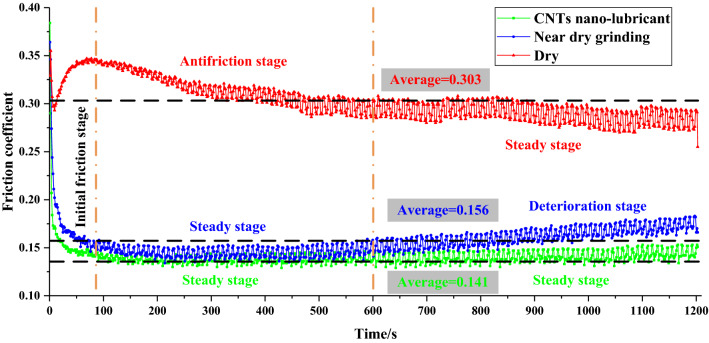
Friction coefficient curve under different lubrication conditions. Figures were created using the Microsoft Visio sofware (version 2010) and Origin (version 2017).

The variation curve of friction coefficient with time under different lubrication conditions is shown in Fig. 3. On the whole, the average friction coefficient of MQL (0.156) and CNT nano-lubricant (0.141) was significantly lower than that of dry condition (0.303), decreasing by 48.51% and 53.47%, respectively. For dry condition, in the initial stage, the friction coefficient decreased sharply and then increased sharply because the surface of the CFRP plate was smooth at first, but then the diamond grinding ball destroyed the surface of the CFRP plate and worsened the contact condition. In the second stage (antifriction stage), the friction coefficient decreased gradually because the friction removal process of the diamond ball on the CFRP plate tended to be stable gradually, and the removed fiber debris acted as a solid lubricant between the interfaces. In the third stage (stable stage), the contact state between the friction pairs tended to be stable, and the diamond grinding ball no longer had a cutting effect on the CFRP plate. For MQL and CNT nano-lubricant conditions, in the initial stage, the friction coefficient decreased sharply under the action of the lubricant. Afterward, the lubricant entered the stable stage and played a full role in lubrication between the friction pairs. The difference was that with the continuous grinding of diamond ball and CFRP plate, the friction coefficient of MQL increased slowly in the third stage, and the frictional contact condition deteriorated because bio-lubricants were insufficient to form an effective lubricant film. In contrast, the friction coefficient curve of the CNT nano-lubricant condition was stable, indicating that the frictional contact state was stable all the time. This condition was due to the fact that CNT nanoparticles played an excellent role in anti-wear and friction reduction between the friction pairs, thus improving the tribological properties and the lasting friction reduction ability of biological lubricants.

### Micromorphology of chips and grooves

**Figure 4:**
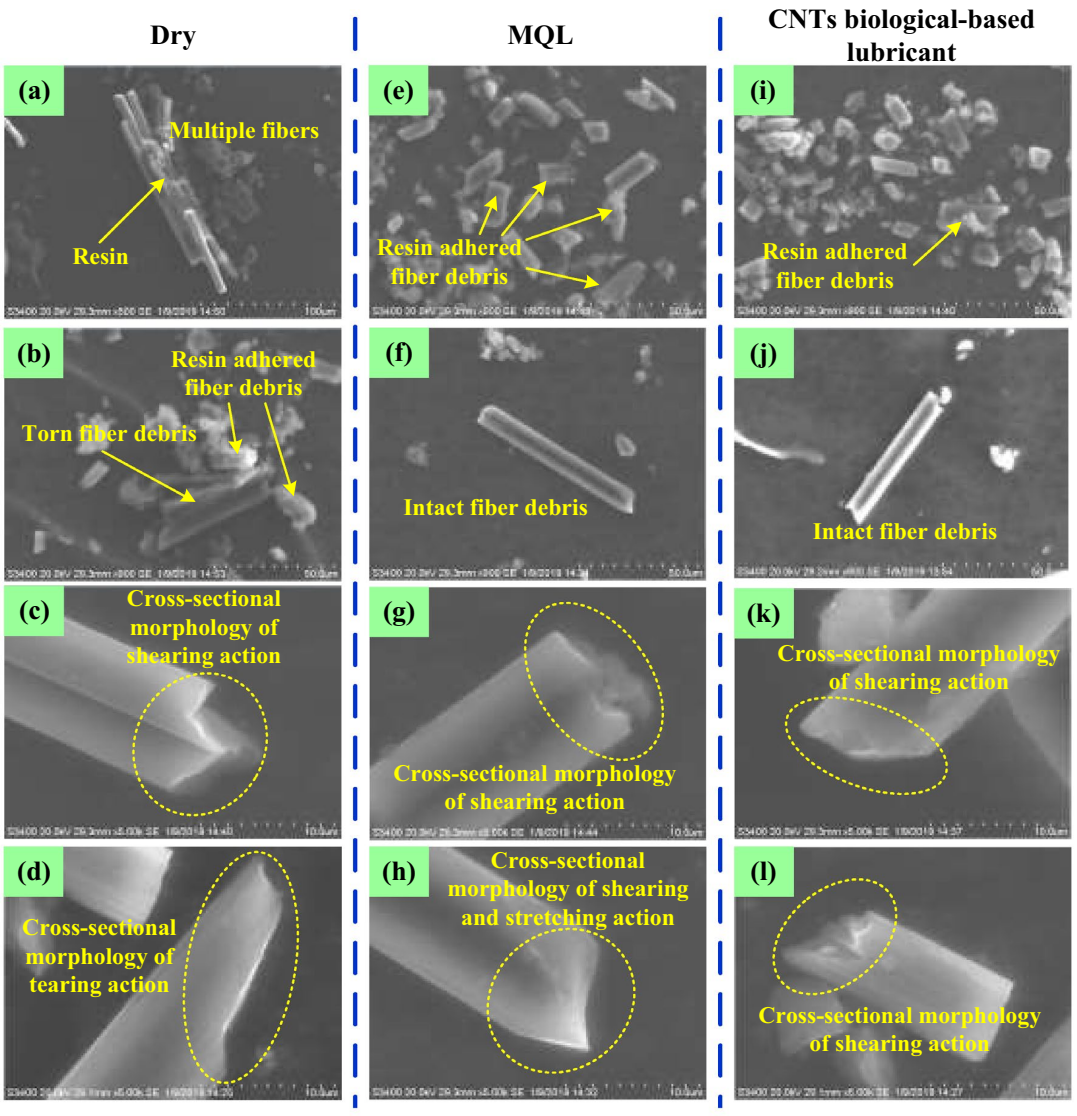
Morphology of grinding debris under different lubrication conditions. Figures were created using the Microsoft Visio sofware (version 2010).

Figure 4 shows the morphology of debris under different lubrication conditions, and block debris of multiple fiber (Fig. 4a) and a large amount of fiber debris adhered by resin (Fig. 4b) during dry grinding can be observed. This finding shows that the binding and supporting effect of the resin matrix and its surrounding materials on the fiber decreased sharply under the influence of grinding temperature under dry condition, resulting in larger cross-scale removal. However, a few adherent debris were found under MQL and CNT nano-lubricant, and block debris with multiple fibers were not found (Fig. 4e,i). Fiber debris under MQL and CNT nano-lubricant appeared as complete rods (Fig. 4f,j), while dry grinding produced broken fibers (Fig. 4b). Shear fracture (Fig. 4c,g,k,l) occurred under three grinding conditions. Tensile fracture and tensile-shear fracture were produced in dry and MQL grinding (Fig. 4d,h).

**Figure 5:**
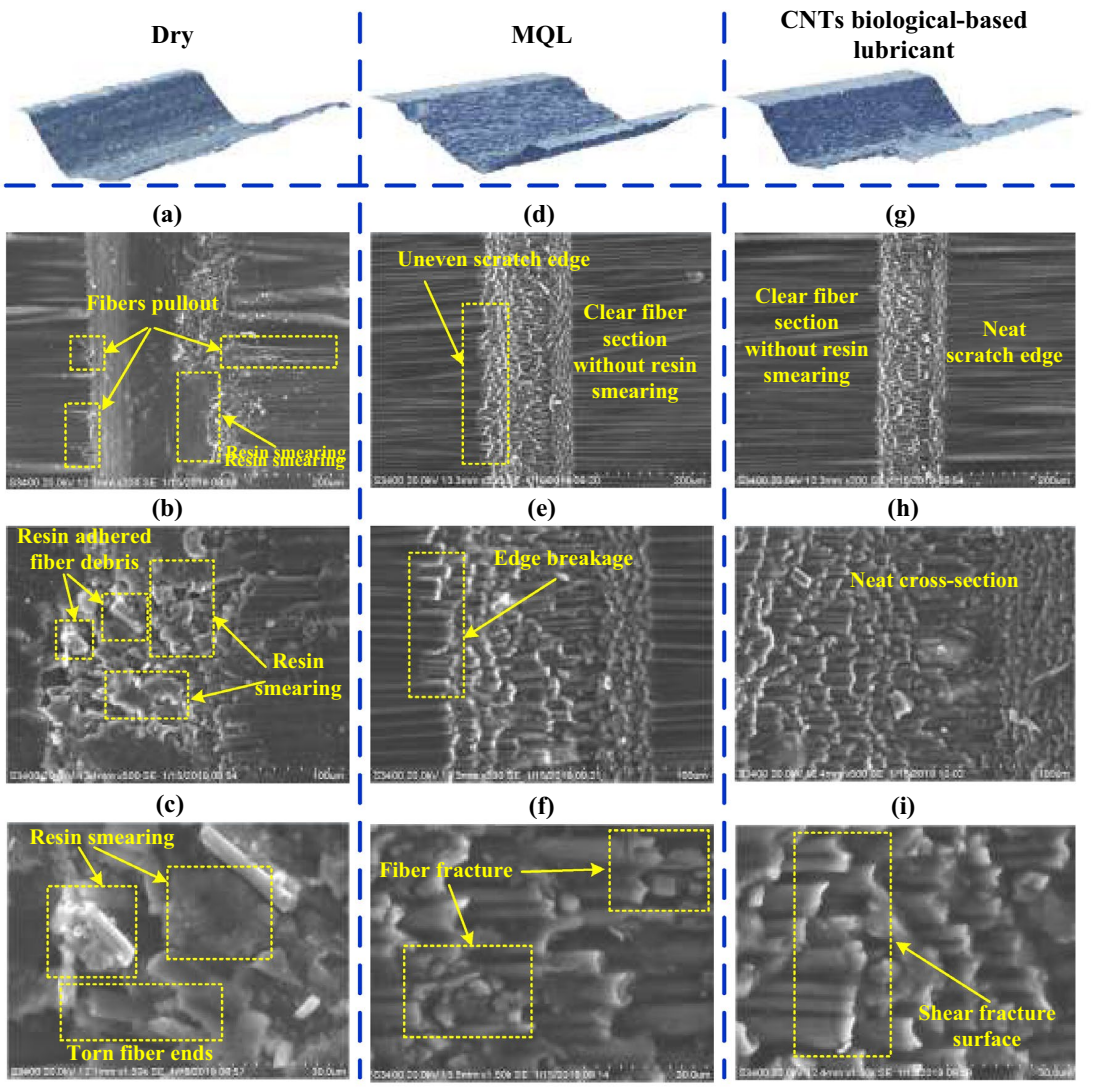
Micromorphology comparison of grinding groove under different lubrication conditions. Figures were created using the Microsoft Visio sofware (version 2010).

As shown in the 3D stereogram in Fig. 5, the grinding groove morphologies were significantly distinct under different lubrication conditions. Under the dry condition, the grinding groove was seriously smeared by the resin and debris. The SEM micrographs in Fig. 5a–c show this phenomenon more clearly. This phenomenon is caused by the resin softening and adhering the fiber debris due to the high grinding temperature, and then smearing the debris on the groove under grain compression. In addition, the edge of the groove was uneven under dry grinding, which was caused by serious fiber pull-out. However, the groove sections under the MQL and CNT nano-lubricant were clearly visible without smearing, especially for the CNT nano-lubricant condition. Fiber pull-out also occurred at the groove edge in dry grinding (Fig. 5a) because of the decreased binding capacity and the supporting effect of resin matrix and surrounding fibers due to the rise of the grinding temperature. Notably, no fiber pull-out was observed in MQL and CNTs nano-lubricants, as shown under the same SEM magnification. However, collapse edge of the groove and fiber breakage occurred under MQL (Fig. 5e,f) in contrast to the CNT nano-lubricant, which had a neat groove edge and fiber section (Fig. 5d,g,h,i). The above phenomenon has two possible reasons. On the one hand, CNTs improved the heat transfer ability of nano-lubricants, which reduced the softening on resin. This condition enhanced the binding ability of the resin matrix and surrounding materials to the fibers to be ground and reduced or avoided edge collapse and fiber pull-out. On the other hand, this phenomenon may be due to the CNTs embedded in the cracks between the removed fiber and the resin matrix, which also played a role in restraining the fibers to be removed. The CNTs are beneficial to the grinding of fiber by diamond grain, which prevented the occurrence of fiber pull-out and edge collapse.

### Surface roughness and morphology

**Figure 6:**
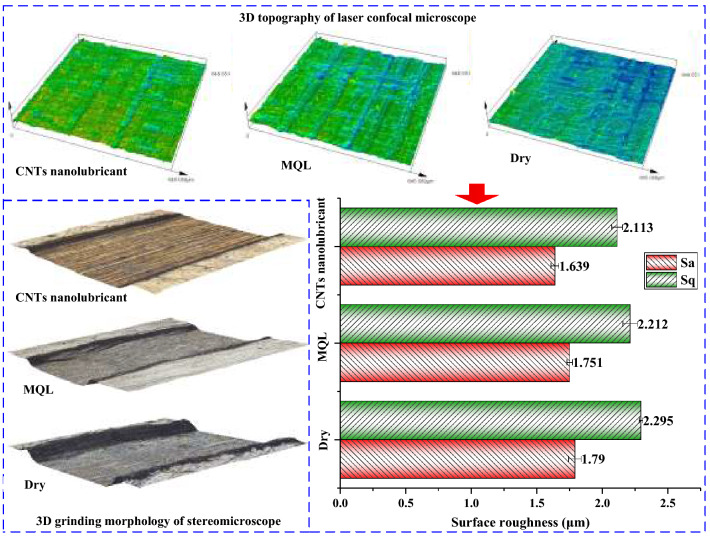
3D morphology and surface roughness of grinding under different lubrication conditions. Figures were created using the Microsoft Visio sofware (version 2010) and Origin (version 2017).

Different from the characterization of two-dimensional surface topography, the characterization of three-dimensional surface topography is a unified parameter that is defined according to the limited scale surface type and does not use three groups of different parameters to characterize the profile, waviness, and roughness respectively. For example, root mean square deviation is the only parameter in the three-dimensional parameters, and its specific meaning depends on the type of finite scale surface used in filtering, rather than the profile parameter *P*_q_, waviness parameter *W*_q_, and roughness parameter *R*_q_. *S*_a_ and *S*_q_ are the parameters that express the amplitude characteristics of surface roughness, and the calculation principle is the same as that of the two-dimensional parameters. However, the range of three-dimensional parameters is a plane area, and the range of points is no longer limited to a certain contour. *S*_a_ represents the arithmetic mean value of the absolute height values of the points on the measured surface and the reference plane in the sampling area. It can generally describe the height deviation of the surface and is used to describe the adhesion and material removal rate of the composite surface after machining. *S*_q_ is the standard deviation of surface height distribution, which is more significant than *S*_a_ in reflecting the distribution of parameter height and characterizing roughness. The results prove that the *S*_a_ and *S*_q_ of dry machining are the largest, and the surface quality was the worst, as shown in Fig. 6. The *S*_q_/*S*_a_ was also large, reaching 1.282, which indicated that the peak or valley height of the surface was relatively obvious. In other words, the surface limit value was obvious. Compared with the results of dry cutting, *S*_a_ and *S*_q_ of MQL were reduced by 2.2% and 3.6% respectively. Compared with the results of dry grinding, *S*_a_ and *S*_q_ of CNT biological lubricant were reduced by 8.4% and 7.9%, respectively. The root mean square deviation *S*_q_ of the workpiece surface in CNT biological lubricant was small, which indicated fewer wave crest features. This condition was due to fewer fiber burrs or protuberances on the surface. The value of each surface roughness is obtained by averaging the five groups of measured values. The standard deviation is calculated according to the formula below

1 $$\sigma { = }\sqrt {\left( {\left( {{\text{x}}_{1} - x} \right)^{2} + \left( {{\text{x}}_{2} - x} \right)^{2} + \cdot \cdot \cdot \cdot \cdot \cdot + \left( {{\text{x}}_{n} - x} \right)^{2} } \right)/n}$$

where *σ* is the standard deviation, *x* is the average of five groups of surface roughness, *x*_1_, *x*_2_, ∙ ∙ ∙ *x*_n_ are the surface roughness values for all, *n* is the amount of groups of surface roughness, which is 5.

### Grinding force of single grain

**Figure 7:**
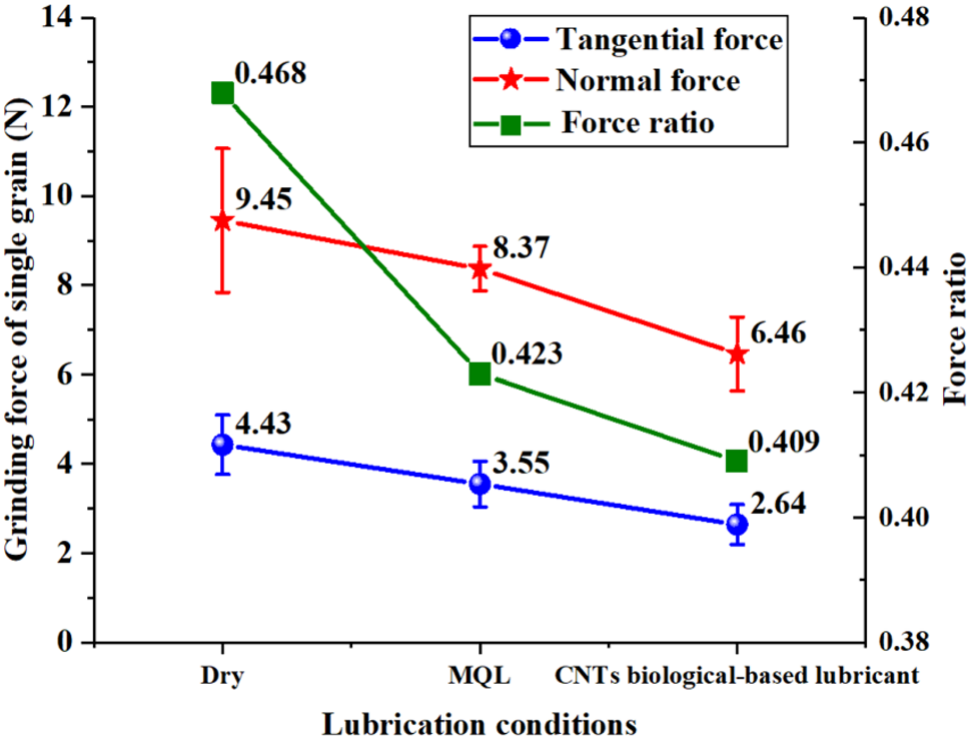
Grinding force and force ratio of single grain grinding under different lubrication conditions. Figures were created using the Origin (version 2017).

**Figure 8:**
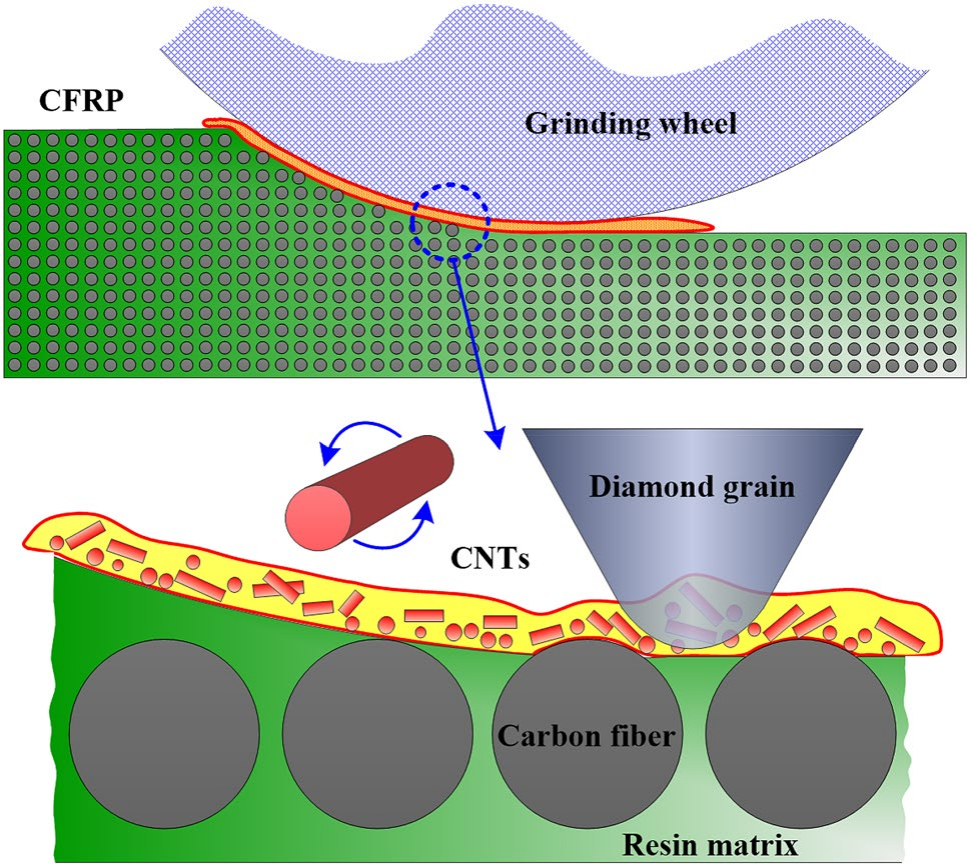
Grinding force and force ratio of single grain grinding under different lubrication conditions. Figures were created using the Microsoft Visio sofware (version 2010).

Five groups of grinding force data were measured and averaged, as shown in Fig. 7. Compared with the results of dry grinding, the specific tangential grinding force and the specific normal grinding force were reduced in different degrees. Under the MQL condition, the tangential grinding force was 3.55 N, the normal grinding force was 8.37 N, and the grinding force ratio was 0.423, thus indicating reductions of 19.86%, 11.43%, and 9.62%, respectively, compared with dry grinding. Under the CNT biological lubricant condition, the tangential and normal grinding force were 2.64 and 6.46 N, respectively, and the grinding force ratio was 0.409, thus indicating reductions of 40.41%, 31.64%, and 12.61%, respectively, compared with dry grinding. From the comparison of the grinding force of single grain, the lubricant formed an effective oil film between the grain and CFRP, which had an obvious lubrication effect. In addition, the cutting effect of diamond grain can be improved by reducing the adhesion of fiber and resin debris. In particular, the bio oil-based CNT nano-lubricants had excellent tribological properties between diamond grain and CFRP, as shown in Fig. 8. CNTs are mainly composed of hexagonal carbon atoms arranged in several layers to dozens of layers of coaxial tubes. As a result of the SP^2^ hybridization of carbon atoms in CNTs, the *S* orbital composition in SP^2^ hybridization was larger than that in SP^3^ hybridization, thus giving CNTs high modulus and strength^57^. CNTs have a special tubular structure and high toughness, which is why they have excellent lubrication and wear resistance. The excellent tribological properties of CNTs may be embodied in the bearing-like effect, which turns the useless sliding friction between grains and fibers into rolling friction.

### Specific grinding energy

**Figure 9:**
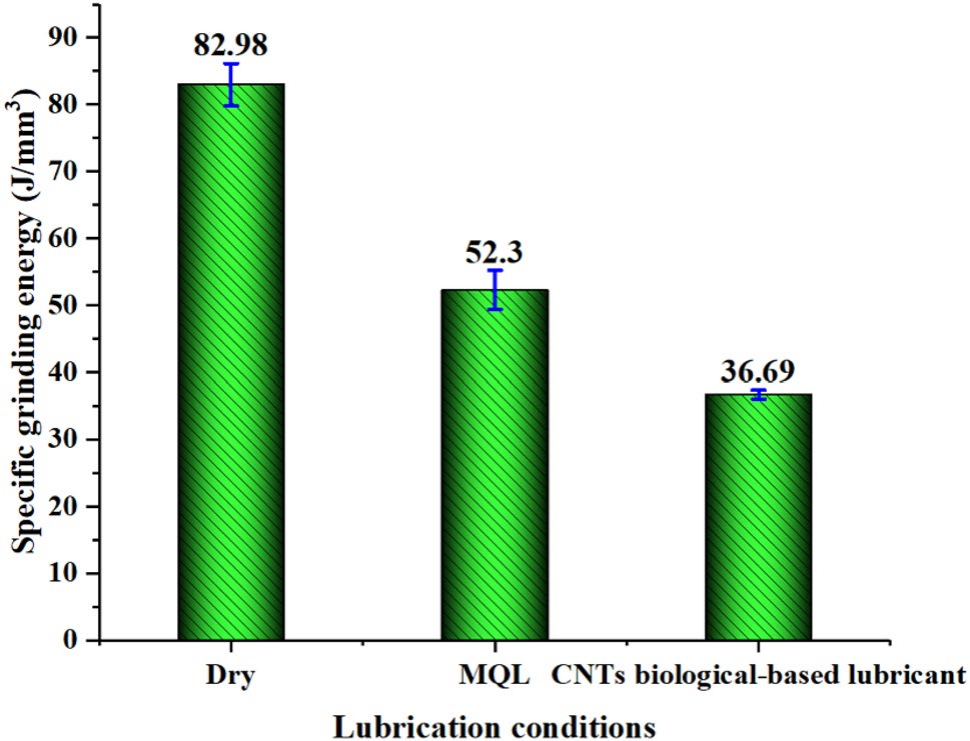
Specific grinding energy of different lubrication conditions. Figures were created using the Origin (version 2017).

Specific grinding energy is an important performance parameter of the grinding process and is defined as the energy consumed by material removal per unit volume. Specific grinding energy is much higher than the specific energy in other machining processes. Specific grinding energy has three components: specific chip formation energy, specific sliding energy, and specific plowing energy^58^. The specific chip formation energy or shear energy formed by the chip is the minimum grinding energy required for material removal. The specific sliding energy is consumed due to the sliding action of the blunt flat tip of the abrasive particles on the surface. Specific plowing energy refers to the energy consumed in the plowing process of sand and stone. The plowing process replaces the material instead of removing it. It mainly reflects the energy required by the plastic plow in the grinding process, which is one of the important indexes to evaluate the grinding efficiency. The low specific grinding energy indicates a high workpiece processing efficiency. The formula of specific grinding energy is as follows:

2 $$U = \frac{P}{{Q_{w} }} = \frac{{F_{t} \cdot v_{s} }}{{v_{w} \cdot a_{p} \cdot b}}$$

where *U* is the specific grinding energy (J/mm^3^), *P* is the total energy consumed by grinding (J), *Q*_*w*_ is the total volume of workpiece material removed, *F*_t_ is the tangential grinding force (N), *v*_*s*_ is the speed of the grinding wheel (m/s), *v*_*w*_ is the workpiece feed speed (mm/s), *a*_*p*_ is the grinding depth of the grinding wheel (mm), and *b* is the workpiece width (mm). As shown in Fig. 9, the specific grinding energy under different lubrication conditions was compared. The specific grinding energy of MQL and CNT biological lubricant was 35.89% and 55.78% lower than that of dry grinding, respectively.

**Figure 10:**
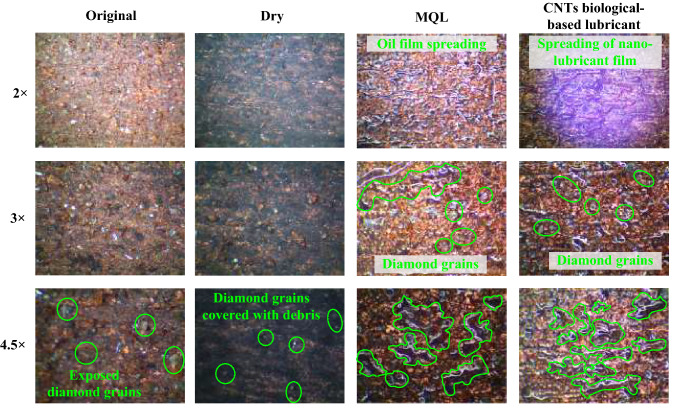
Comparisons of grinding wheel blockage under different lubrication conditions. Figures were created using the Microsoft Visio sofware (version 2010).

The lowest specific grinding energy was obtained under CNT biological lubricant condition because of the minimum tangential force. The essential reason for this situation was that CNT biological lubricants play an effective role in reducing friction between the grinding wheel and the CFRP plate. A more important detail is that the new lubrication method greatly inhibited wheel clogging. Wheel blockage usually results in a sharp increase in grinding force and specific grinding energy, and poor machining quality^59^. The comparison of wheel clogging and oil film on the grinding wheel surface during grinding CFRP under different conditions is shown in Fig. 10. Serious grinding wheel clogging in dry grinding was observed. The grinding wheel surface of MQL and CNT biological lubricant was relatively clean, no adhesion blockage of a large number of fiber debris occurred, and obvious oil film spreading and covering were found on the grinding wheel surface, which was conducive to lubricating the grinding wheel workpiece interface and reducing the grinding wheel blockage. The reason for this condition is that the high grinding temperature of dry grinding leads to the softening of the resin matrix. The softened resin matrix adheres to the grains and bonds with the fiber debris, which would accumulate with the grinding process. In addition, under the dry grinding condition, the grain wear on the grinding wheel was serious, and the contact area between the grinding wheel and the workpiece increased, thereby increasing the difficulty of removing the debris and, in turn, worsening the adhesion of the debris. In MQL and CNT biological lubricant grinding, the oil film of MQL oil and nano-lubricant between the grinding wheel and workpiece interface can effectively reduce and avoid the blockage of grinding chips on the grinding wheel. Moreover, the grinding temperature of MQL and CNT biological lubricant conditions was low, which prevented the thermal softening of resin matrix, and few fiber debris adhered to the grinding wheel surface. The high-pressure gas from the nozzle also had a certain cleaning effect on the grinding wheel surface, which could remove the debris blocked by the attached and embedded grinding wheels, further reducing the blockage on the surface of the grinding wheel and improving the service life of the grinding wheel.

## Conclusions

In this paper, the grindability and frictional behavior of the new lubrication method of CNT biological lubricant were evaluated by performing friction-wear tests and single grain and grinding wheel experiments under different lubrication conditions. The chip morphology, groove morphology and grinding force of single grain, three-dimensional surface roughness, specific grinding energy, and wheel clogging were evaluated. The following conclusions were obtained:

1. Compared with the dry condition, MQL and CNT biological lubricant significantly reduced the friction coefficient by 48.51% and 53.47%, respectively. In addition, CNT nano-lubricants showed optimal and durable antifriction properties.
2. MQL and CNT biological lubricants were beneficial to suppressing the removal of multifiber block debris during grinding. In addition, CNT biological lubricant reduced the occurrence of tensile fracture and tensile shear fracture.
3. *S*_a_ and *S*_q_ of CNT biological lubricant were reduced by 8.4% and 7.9%, respectively, compared with dry grinding, indicating that the wave crest features were fewer, which was due to fewer fiber burrs or protuberances on the surface.
4. Under the CNT biological lubricant condition, the tangential and normal grinding force of single grain were 2.64 and 6.46 N, respectively, and the grinding force ratio was 0.409, thus indicating reductions of 40.41%, 31.64%, and 12.61%, respectively, compared with dry grinding. The specific grinding energy of MQL and CNT biological lubricant was 35.89% and 55.78% lower than that of dry grinding, respectively.
5. Dry grinding caused severe wheel blockage. MQL and CNT biological lubricants can effectively improve the surface condition of the grinding wheel through temperature control, chip removal, and oil film formation. The addition of CNTs nanoparticles is conducive to the formation and spread of oil film.
